# From pathophysiology to novel approaches for obesity-associated hypertension

**DOI:** 10.1093/ckj/sfaf218

**Published:** 2025-07-09

**Authors:** Mehmet Kanbay, Elif Yayci, Candan Genc, Sidar Copur, Ozgur Aktas, Pantelis Sarafidis, Adrian Covic, Alberto Ortiz, Luke J Laffin

**Affiliations:** Department of Medicine, Section of Nephrology, Koç University School of Medicine, Istanbul, Turkey; Department of Medicine, Koç University School of Medicine, Istanbul, Turkey; Department of Medicine, Koç University School of Medicine, Istanbul, Turkey; Department of Medicine, Koç University School of Medicine, Istanbul, Turkey; Department of Medicine, Koç University School of Medicine, Istanbul, Turkey; First Department of Nephrology, Hippokration Hospital, Aristotle University of Thessaloniki, Thessaloniki, Greece; Nephrology Clinic, Dialysis, and Renal Transplant Center, “C.I. Parhon” University Hospital, Iasi, Romania; Department of Medicine, Universidad Autonoma de Madrid and IIS-Fundacion Jimenez Diaz, Madrid, Spain; Section of Preventive Cardiology and Rehabilitation, Department of Cardiovascular Medicine, Cleveland Clinic Foundation, Cleveland, OH, USA; Cleveland Clinic Coordinating Center for Clinical Research, Cleveland Clinic Foundation, Cleveland, OH, USA

**Keywords:** adiposity, bariatric surgery, GLP-1 receptor agonists, hypertension, obesity

## Abstract

Obesity is a rapidly growing epidemic affecting >15% of the global adult population and has considerable clinical consequences and comorbidities, including hypertension, diabetes mellitus, cardiovascular and cerebrovascular diseases and chronic kidney disease. There is a strong association between obesity or body mass index and high blood pressure (BP) in epidemiological studies while the underlying pathophysiological events linking those conditions are not fully elucidated. Hypothetical mechanisms include a sedentary lifestyle and excess intake of processed foods that contribute to obesity, overactivation of the renin–angiotensin–aldosterone and sympathetic nervous systems, inflammation, altered adipokine homeostasis and the fatty kidney hypothesis involving adipose tissue accumulation in the renal sinus and perirenal space. There are multiple pharmacotherapeutic and surgical approaches for the management of obesity, including dual and triple agonist drugs targeting glucagon-like peptide-1, gastric inhibitory peptide and glucagon receptors and endoscopic bariatric procedures. Despite promising results with such therapeutic approaches in terms of body weight reduction and BP control, it is unclear whether such BP reduction may completely be attributable to weight loss. Confirmation of the adiposity dependence would lead to a major paradigm shift in our understanding of hypertension, potentially leading to a major shift in the causes of hypertension from primary hypertension to adiposity-dependent hypertension, leading to a shift from symptomatic treatment with antihypertensive medication to cause-focused treatment with weight loss medication. In this narrative review, the aim is to evaluate the potential pathophysiological mechanisms linking hypertension and obesity and the efficiency of potential therapeutic approaches on BP.

## INTRODUCTION

Obesity, a growing epidemic defined as a body mass index (BMI) ≥30 kg/m^2^ according to the World Health Organization definition, affects >15% of the global adult population and led to an estimated five million deaths globally in 2019 [1, 2]. Obesity is linked to multiple non-communicable diseases, including hypertension, cardiovascular and cerebrovascular diseases, diabetes mellitus, hyperlipidaemia, chronic kidney disease, obstructive sleep apnoea and malignancies [3]. Obesity has been implicated as the cause for hypertension in 26% of males and 28% of females according to the Framingham Heart Study [3, 4]. Moreover, weight gain is associated with elevated blood pressure (BP) while high BP also leads to considerable deterioration in obesity-related comorbidities, including cardiovascular and cerebrovascular events and chronic kidney disease [5]. Therapeutic control of hypertension is more challenging in patients with obesity or overweight in addition to a higher disease burden [6, 7]. Weight loss is a fundamental part of BP control in individuals with overweight and obesity and novel obesity medications and endoscopic bariatric procedures have shown promising results in clinical trials [8, 9]. Moreover, obesity is usually preceded by being overweight, a condition that is treatable by anti-obesity medications. Treatment of overweight is an indication approved by regulatory agencies when it coexists with hypertension (https://www.ema.europa.eu/en/medicines/human/EPAR/wegovy). Understanding the underlying pathophysiological events linking obesity and high BP are crucial, as it directly implies the utilization of various pharmacotherapeutic options. Current strategies to treat overweight and obesity are promising, however, there is limited understanding of how they achieve BP reductions. This review aims to describe the mechanisms of hypertension in obesity and explore the effects of different obesity treatment strategies in reducing BP.

## THE PATHOPHYSIOLOGICAL PERSPECTIVE

**Figure 1:**
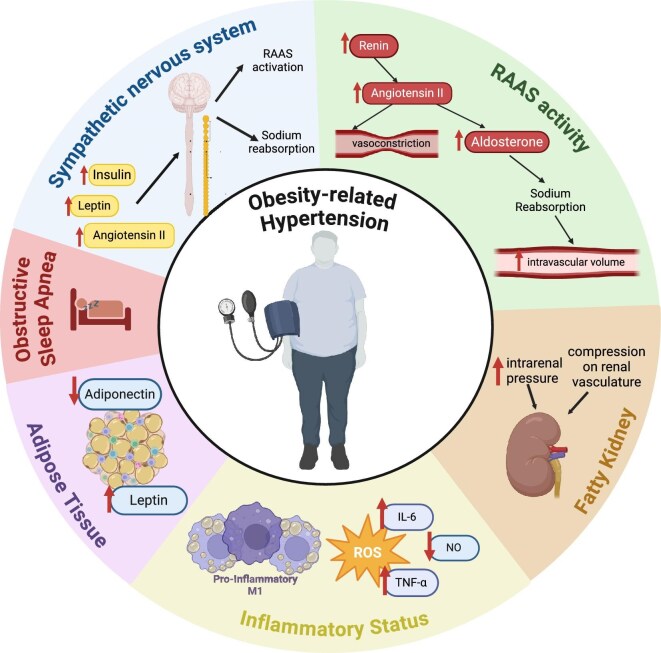
The potential underlying pathophysiological mechanisms of obesity-related hypertension. ROS: reactive oxygen species.

There are multiple underlying pathophysiological mechanisms potentially linking obesity and high BP (Fig. 1).

### Renin–angiotensin–aldosterone system (RAAS)

Activation of the RAAS has been proposed as a mechanism leading to high BP among patients with obesity. In addition to the elevated plasma levels of renin, angiotensin II, angiotensin-converting enzyme (ACE), angiotensinogen and aldosterone, renin receptors are also upregulated in patients with obesity compared with lean subjects, all of which may contribute to obesity-mediated hypertension [4]. Both RAAS activation and upregulation of angiotensin II are involved in systemic vasoconstriction and aldosterone-mediated increased tubular water and sodium retention. Under physiological conditions, such sodium retention and volume expansion should suppress the RAAS via negative feedback mechanisms while such regulatory mechanisms are impaired in patients with obesity. RAAS activation in patients with obesity is driven mainly by two mechanisms: physical compression due to fat accumulation, referred to as the fatty kidney hypothesis, and increased sympathetic nervous system activity. Moreover, adipocytes are potential contributors to excess angiotensinogen, angiotensin II and aldosterone production [10]. Angiotensin II contributes to obesity-related hypertension by stimulating renal sodium chloride reabsorption. Additionally, angiotensin II is a strong vasoconstrictor, and by constricting efferent arterioles, it causes increased glomerular hydrostatic pressure, which can be explained as the intrarenal effect [11]. Understanding the impact of the RAAS in obesity-related hypertension is important for understanding and improving treatments. Such pathophysiological significance of RAAS overactivity among obesity-mediated hypertension has clinical significance as well. Pharmacotherapies targeting RAAS, including ACE inhibitors, angiotensin receptor blockers, mineralocorticoid receptor antagonists (MRAs) or renin inhibitors, may decrease high BP among patients with obesity [12, 13]. However, they are also effective antihypertensive agents for primary hypertension in general. The fact that hypertension in obese individuals is notoriously difficult to control argues against a key, obesity-specific role of the RAAS that differs from its role in non-obese individuals.

### Fatty kidney hypothesis

Fatty kidney is a recently evaluated concept in clinical research, linked to intrarenal, perirenal and renal sinus fat accumulation [14]. Such adipose tissue accumulation leads to kidney injury and metabolic dysfunction through various mechanisms including inflammation and oxidative stress, Klotho deficiency, mitochondrial dysfunction, endoplasmic reticulum stress and RAAS activation mediated by the mechanical pressure induced by the perirenal and renal sinus fat tissue [15]. Fat accumulation in the renal sinus may negatively impact renal function by physical compression and by hormonal and inflammatory mechanisms that can lead to decreased renal function and increased BP [16]. Increased fat tissue in the renal sinus activates RAAS by compressing renal vasculature or by depolarizing nerves lining the vessels [15]. Also, renal parenchymal fat accumulation leads to inflammation and oxidative stress, causing the release of pro-inflammatory cytokines that damage the renal vasculature and structure [17].

### Sympathetic overactivation

Sympathetic nervous system (SNS) activity is increased in patients with obesity and is thought to contribute to obesity-related hypertension [18, 19]. Several factors contribute to this increased SNS activation: baroreceptor reflex impairment (impairment of baroreceptor control on sympathetic nerve activity) [20]; hyperinsulinemia (insulin activates the SNS directly through hypothalamic action) [21]; angiotensin II, along with other RAAS metabolites, contributes to increased SNS activation; cytokine release from adipocytes, especially leptin, is a key contributor, which will be detailed below; and the pro-opiomelanocortin (POMC) pathway.

Although obesity induces overactivation of the SNS and downregulation of the parasympathetic nervous system, such an effect on the SNS is mostly mild and not applicable to all tissues, as increased heart rate associated with obesity is mostly attributable to downregulation of parasympathetic nervous system [22, 23]. Nonetheless, SNS overactivation is evident at in the renal and musculoskeletal system in patients with obesity [24, 25]. Despite these observations, there is no therapeutic strategy in routine clinical use targeting the SNS in obesity-associated hypertension.

### Adipokines

Obesity is associated with an altered homeostasis of adipokines, i.e. bioactive molecules produced by adipocytes. Leptin and adiponectin are the most important adipokines that have been linked to BP regulation.

Circulating leptin levels positively correlate with BP in obesity [26], and leptin can increase BP via SNS activation [22]. Leptin resistance in obesity impacts appetite and satiety, but it does not modify the effect of leptin on the SNS [27]. Leptin activates the SNS via the melanocortin 4 receptor (MC4R), a receptor regulated by POMC [5, 28]. In leptin-deficient animals and individuals with leptin deficiency, sympathetic activation, RAAS activity and BP were lower [29–31].

Adiponectin protects from high BP and other cardiovascular events. Adiponectin mediates vasodilation via nitric oxide (NO) production in endothelial cells [26]. It also has anti-inflammatory functions, decreasing oxidative stress, tumour necrosis factor α (TNF-α) levels and macrophage activity [32]. Indeed, lower adiponectin levels are associated with hypertension [33].

Overall, despite preclinical evidence and associations observed in the clinic, a putative role of adipokines in clinical obesity-associated hypertension remains uncharacterized due to the lack of interventional studies specifically targeting these molecules.

### Pro-inflammatory status

Obesity is a state of inflammation with high levels of circulating pro-inflammatory cytokines [34]. TNF-α, interleukin-6 (IL-6) and plasminogen activator inhibitor-1 (PAI-1) may regulate BP in obesity. Higher TNF-α and IL-6 levels are positively correlated with BMI and negatively correlated with BP levels [26]. TNF-α reduces NO-mediated vasodilation and influences endothelin-1 levels [35]. Association studies have suggested a potential IL-6 role in elevated BP in obesity via angiotensin II, which needs to be confirmed by interventional studies [36, 37]. In addition to inflammatory cytokines, adipose tissue macrophages further contribute to the inflammatory state, especially pro-inflammatory M1 macrophages [38].

Despite the presence of overwhelming preclinical and clinical data linking pro-inflammatory status with obesity-associated hypertension, there are currently no clinical data illustrating beneficiary effects of any anti-inflammatory drugs on obesity-associated hypertension.

### Obesity-associated lifestyle

Obesity is frequently a consequence and cannot be dissociated from a lifestyle characterized by different combinations of sedentarism, excess food intake and excess ingestion of processed foods. Exercise has both acute and chronic BP-lowering effects, and when associated with sweating may contribute to sodium losses. Processed foods are usually rich in salt. Indeed, one mechanism of the anti-obesity effect of glucagon-like peptide-1 (GLP-1) receptor agonists is satiety, i.e. reduced food intake and all its components such as salt. We should not underestimate the contribution of a lifestyle that facilitates the development and maintenance of obesity to the association of obesity with hypertension.

## MECHANISMS OF UNCONTROLLED AND RESISTANT HYPERTENSION

In addition to being associated with a higher risk for hypertension, obesity is associated with impaired response to pharmacotherapy or lifestyle interventions [6]. Sympathetic activation is suggested to contribute to sodium retention via thiazide-sensitive sodium chloride co-transporter (NCC) upregulation as observed in obese Zucker rats [39]. However, a dynamic role of sodium transporters in obesity-associated hypertension has been reported. NCC contributes to sodium overload in the early stages of obesity and increased sodium–potassium–2 chloride co-transporter (NKCC2) activity in later stages, implying a possible need to target different transporters according to the course of obesity [40].

Treatment non-adherence is another crucial aspect of hypertension control. Obesity is a risk factor for non-adherence to lifestyle or pharmacological interventions [41]. Ensuring patient compliance should be prioritized during hypertension management.

Resistant hypertension is defined as failure to reach the target BP despite the use of three antihypertensive drugs from different classes, one of them being a diuretic, at maximum doses. The mechanism of resistant hypertension in obesity is multifactorial, with interactions of the nervous system, hormones, adipokines and non-compliance [42]. Excess aldosterone production by adipocytes has been proposed as a mechanism of uncontrolled hypertension in obesity. MRAs are recommended in cases of drug-resistant hypertension in all patients, including those with obesity. In salt-sensitive hypertension, aldosterone levels are not reduced with exposure to high amounts of sodium despite RAAS inhibition [43]. As a result, MRAs may improve BP in salt-sensitive hypertension. More recently, selective aldosterone synthase inhibitors have been shown to safely lower BP in a population with a mean BMI of 32 kg/m^2^ [44, 45]. Importantly, in preclinical studies, MRA activation is key for salt-sensitive hypertension [46, 47]. Multiple studies have demonstrated that the amount of dietary sodium intake has significantly more impact on BP in obesity compared with a normal BMI, underlying the importance of sodium intake for regulation of BP [48, 49]. While medications are important to regulate BP, proper diet is crucial in obesity-associated hypertension and difficult to achieve given that improper diet is a root cause of obesity generation and maintenance.

## IMPACT OF OBESITY ON THE EFFICACY OF ANTIHYPERTENSIVE MEDICATIONS

The major guidelines of hypertension treatment in obese individuals recommend weight loss; however, they do not discuss specific antihypertensive medications in these patients [50]. Specific characteristics of individuals with obesity-related hypertension influence the effectiveness of antihypertensive medications.

First, pharmacokinetic and pharmacodynamic properties of antihypertensive medications may vary in individuals with different BMI categories. Obesity may change the pharmacokinetics of medications due to an increased volume of distribution and decreased levels of active pharmaceuticals in the standard doses [51], including for certain antibiotics, anticoagulants and some antineoplastic medications [52, 53]. However, antihypertensive medications are less well characterized. Altered activity of propranolol and atenolol [54] and higher responsiveness of BP to α-adrenergic inhibition have been reported in obesity [55]. In a preclinical study, intravenous nebivolol had a higher total clearance and distribution volume among obese participants without differences in half-life [56]. Altered gastrointestinal transit and absorption, hepatic first-pass metabolism and drug metabolism may affect drug bioavailability of antihypertensive medications in patients with high BMI, while tissue distribution is a major concern, especially for highly lipophilic medications. Furthermore, considerable changes in CYP enzyme bioactivity, including low CYP3A and CYP2C19-mediated metabolism and increased CYP2E1 and CYP2D6-mediated metabolism, may alter the half-life of various drugs [57].

Second, some pathophysiological processes are reported to be more activated in obesity-related hypertension than in non-obese individuals. These include increased sympathetic nervous system activity, a decrease in baroreceptor sensitivity, perivascular adipose tissue accumulation leading to endothelial dysfunction and vasoconstriction, an increase in leptin and pro-inflammatory cytokines, fatty kidney leading to overactivity of the RAAS causing volume expansion and renal hyperfiltration [58]. For instance, the concentration of beta-adrenergic receptors was increased among obese patients and decreased with weight loss [59, 60]. While it may be hypothesized that antihypertensive medications targeting such pathophysiological processes may be more effective in BP regulation in obese patients, this appears not to be the case, given that resistant hypertension is common in obesity [6]. In this regard, a meta-analysis involving a total of 135 715 patients from 22 clinical trials failed to identify a superiority of any antihypertensive drug group on cardiovascular risk except for slightly better protection with ACE inhibitor therapy compared with calcium channel blockers for each 5 kg/m^2^ increase in BMI [61]. Moreover, the potential role of anti-obesity pharmacotherapeutics on BP control should not be overlooked [62]. Nonetheless, there is a clear need for future large-scale clinical trials evaluating the effectiveness of various antihypertensive drugs versus anti-obesity medications among obese or overweight patients. However, we appreciate the challenges in conducting such a clinical trial. Lastly, physicians may perceive obese patients as less adherent to antihypertensive medications, likely influenced by the lower rates of BP control [63].

## THERAPEUTIC APPROACHES

**Figure 2:**
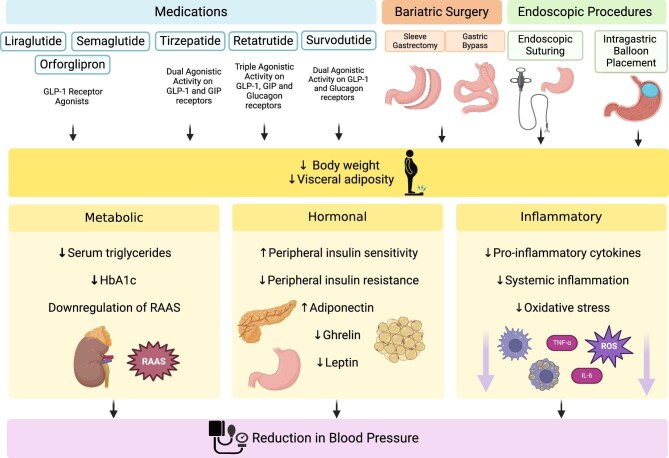
Potential therapeutic options for the management of high BP among obese or overweight individuals. ROS: reactive oxygen species.

Multiple pharmacotherapeutic approaches have been approved by the US Food and Drug Administration for the management of overweight and obesity, including orlistat, a gastric and intestinal lipase inhibitor; liraglutide, semaglutide and tirzepatide; GLP-1 receptor agonists; bupropion/naltrexone or phentermine/topiramate combination [64]. Additionally, the European Medicines Agency has also approved several pharmacotherapeutic options for overweight and obesity, including tirzepatide, semaglutide and liraglutide, highlighting the growing recognition of obesity as a critical medical condition. Moreover, bariatric surgery and endoscopic interventions may offer additional beneficial effects for patients failing to reach the desired weight loss with lifestyle modifications and pharmacotherapies (Fig. 2). Weight loss is a fundamental part of the management of hypertension among patients with obesity or overweight, as illustrated in multiple previous large-scale meta-analysis studies indicating an ≈1 mmHg decline is systolic BP (SBP) per 1 kg decrease in body weight [65], although it is far from being the complete picture of obesity-related hypertension treatment.

### GLP-1 receptor agonists

These include liraglutide and semaglutide. While liraglutide is administered subcutaneously, semaglutide is available in oral and parenteral formulations. Treatment with liraglutide reduced SBP by 2.5 mmHg within 2 weeks of treatment prior to any significant change in body weight [66]. Such improvements in BP control persist over chronic therapy along with a decrease in body weight [67, 68]. Similar effects have also been reported for semaglutide therapy [69]. The SELECT trial (NCT03574597) was a large-scale randomized clinical trial involving 17 604 patients with obesity without diabetes, randomized to semaglutide or placebo. Semaglutide reduced cardiovascular risk [hazard ratio (HR) 0.80, *P* < .001] over a mean follow-up period of 39.8 months [70], including a reduction of major adverse cardiovascular events (MACE), a composite of heart failure, cardiovascular death and all-cause mortality, in patients with or without known heart failure [71]. Therefore, the benefits of semaglutide extend beyond weight and BP reduction to offer cardiovascular and kidney protection [72], two key complications of hypertension. The exact mechanism of BP reduction by GLP-1 receptor agonists remains unclear. Weight and fat loss, reduced salt intake (within a wider context of decreased food intake), attenuation of RAAS activation and potential natriuretic effects may contribute [68]. Orforglipron is a novel once-daily oral alternative that reduced SBP by up to 10.5 mmHg at weeks 26 and 36 compared with 3.6 mmHg and 1.8 mmHg, respectively, with placebo. However, no significant changes were observed in diastolic BP (DBP) [73].

### Dual agonists

These include tirzepatide, a dual agonist of GLP-1 and gastric inhibitory polypeptide (GIP) receptors or twincretin, and survodutide, a dual agonist agent of GLP-1 and glucagon receptors. The beneficial effects of tirzepatide therapy go beyond superior glycaemic control compared with GLP-1 analogue or basal insulin therapy, including reductions in body weight, visceral adiposity and BP, as well as an improved serum lipid profile and kidney outcomes [74, 75]. A subgroup analysis of the SURMOUNT-1 study (NCT04184622) of tirzepatide involving 600 participants undergoing 24-hour ambulatory BP recording revealed a placebo-adjusted mean decrease of 7.4 mmHg in SBP with 5 mg/week, 10.4 mmHg with 10 mg/week and 8.0 mmHg with 15 mg/week. Although pooled analysis indicated a correlation between body weight and BP changes, mediation analysis revealed that BP reductions were only partially attributed to body weight changes {mediated 70.0% [95% confidence interval (CI) 47.0–102.6]} [76]. A 52-week clinical trial showed the superiority of tirzepatide over dulaglutide, a GLP-1 analogue medication, in terms of BP reduction [77]. Most trials of tirzepatide have shown beneficial effects on BP control [74]. Besides decreasing weight and improving peripheral insulin sensitivity, potential contributing factors include increased adipose tissue adiponectin production, reduced pro-inflammatory cytokine and reactive oxygen species release, inhibition of monocyte/macrophage activation and release of vasodilatory substances including NO [74].

Additional activation of glucagon receptors with survodutide may increase energy expenditure, decreasing liver fat stores and metabolic dysfunction–associated fatty liver disease (MAFLD) [78–82]. Survodutide may also decrease BP [80, 82]. A randomized controlled phase 2 clinical trial enrolled 293 biopsy-confirmed MAFLD patients. Over 48 weeks, survodutide reduced SBP by 7.5 ± 13.0 mmHg, 6.55 ± 13.9 mmHg and 4.9 ± 14.3 mmHg at 2.4 mg, 4.8 mg and 6.0 mg doses, respectively. Moreover, it reduced DBP by 2.98 ± 7.83 mmHg, 1.30 ± 9.80 mmHg and 1.33 ± 7.77 mmHg, respectively [80].

### Triple agonists

Retatrutide activates GLP-1, GIP and glucagon receptors. It is under clinical development for the management of metabolic syndrome and obesity. In clinical trials, retatrutide decreased visceral fat and body weight [83, 84]. In a phase 2 placebo-controlled clinical trial that enrolled 338 adults with obesity or overweight and comorbidities, retatrutide also improved cardiometabolic risk factors, including SBP and DBP, over 48 weeks. Overall, 41% of participants on retatrutide 8 mg/week and 30% on 12 mg/week were successfully weaned from at least one antihypertensive medication [85]. A meta-analysis including two large-scale randomized controlled clinical trials found that retatrutide reduced SBP by 7.64 mmHg (95% CI 9.51–5.76) and DBP by 2.33 mmHg (95% CI 3.44–1.23). Strikingly, 8 mg/week (starting dose 4 mg) decreased BP to a greater extent than in the 12 mg/week group despite more substantial weight loss in the latter [86].

### Bariatric surgery

Bariatric surgery is an invasive procedure generally offered to patients with a BMI >40 kg/m^2^ or 35–40 kg/m^2^ along with obesity-related comorbidities, including metabolic or cardiovascular complications, if lifestyle modifications and pharmacotherapeutic approaches fail [87]. Bariatric surgery may decrease weight and potentially improve obesity-related comorbidities as well. A prospective cohort study enrolling 787 patients undergoing either gastric bypass or sleeve gastrectomy procedures with a baseline mean BMI of 44.6 kg/m^2^ investigated their impact on BP control. Bariatric surgery led to remission of hypertension in 84% of patients, defined as normalization of BP along with the discontinuation of antihypertensive therapies, and a relapse rate of 31.4%, defined as the need for new antihypertensive medication or BP control. Weight regain or an inability to lose weight were identified as potential risk factors for hypertension relapse [88]. Another study involving 120 patients undergoing laparoscopic Roux-en-Y gastric bypass and laparoscopic sleeve gastrectomy with a follow-up period of up to 5 years found hypertension remission in 54% [89]. Remission of hypertension was reported in 29% of patients undergoing sleeve gastrectomy and 51% of patients undergoing gastric bypass despite similar weight loss rates (49% versus 57%) [90]. A randomized clinical trial investigated the efficiency of bariatric surgery in addition to medical therapy in patients with grade 1–2 obesity and hypertension requiring at least two antihypertensive medications at maximum doses or more than two antihypertensive medications at moderate doses. Addition of the Roux-en-Y gastric bypass procedure to medical therapy did not improve the 24-hour BP profile compared with medical therapy alone, though it improved nighttime SBP variability and reduced resistant hypertension rates [91]. A meta-analysis of >22 094 patients with a mean baseline BMI of 46.9 kg/m^2^ reported that hypertension resolved in 61.7% of patients and either resolved or improved in 78.5% of patients along with improvements in other obesity-related comorbidities such as diabetes mellitus, hyperlipidaemia and obstructive sleep apnoea [92]. A few large-scale meta-analyses support that bariatric surgery may provide beneficial effects on BP regulation over long-term follow-up [93, 94]. The number of antihypertensive medications used prior to bariatric surgery is a strong predictor of hypertension remission and relapse rates [95]. However, the comparison of bariatric surgery and pharmacotherapeutic approaches is premature from reaching a definite conclusion, as available clinical studies have considerable limitations in terms of study design and clinical follow-up periods. Moreover, the long-term clinical outcomes, including BP regulation, are unclear in both pharmacotherapeutic approaches and bariatric surgeries.

An important consideration when assessing bariatric surgery–related improvements in BP readings is whether it translates to a reduction in MACE or mortality. A review study of the Swedish Obese Subjects trials reported that bariatric surgery reduced the incidence of myocardial infarction [adjusted HR (aHR) 0.71, *P* = .02), stroke (aHR 0.66, *P* = .008) and overall mortality events (aHR 0.71, *P* = .01) compared with conventional therapies [96]. Such results indicate that bariatric surgery reduces cardiovascular risk.

A major consideration is whether bariatric surgery–related BP regulation is attributable to weight loss alone or whether there are other potential confounders. Reduced peripheral insulin resistance, RAAS activation, systemic inflammation and improvements in other obesity-related comorbidities, including hyperlipidaemia and diabetes mellitus, in response to weight loss may contribute to BP regulation following bariatric surgery [97]. Significant BP reduction following bariatric surgery is reported as early as postoperative week 1, indicating potential non-weight loss–related contributors to BP control. In a prospective cohort study involving 100 patients undergoing laparoscopic Roux-en-Y gastric bypass surgery, SBP (−9 mmHg) and DBP (−7 mmHg) decreased within 1 week and the improvement was maintained throughout 1 year of follow-up [98]. Potential underlying mechanisms for such early and non-weight loss–related improvements in BP include reduced salt intake as part of a decrease in food intake. Additionally, incretins such as GLP-1 and peptide YY may increase and induce natriuresis [99] or regulate the SNS through receptors at the area postrema [100]. Modulations of other adipokines, such as ghrelin, leptin or adiponectin, may also contribute to BP regulation following bariatric surgery [101, 102]. Within 1 week of surgery, leptin levels decrease by 50% and adiponectin levels increase and these changes are maintained for at least 1 year [102]. Pro-inflammatory cytokines such as IL-6 and C-reactive protein levels decreased significantly following bariatric surgery, which may contribute to BP regulation [103]. These factors, especially gut-mediated incretins, may account for the differences in BP improvement between various bariatric surgical approaches [98, 104]. However, there is a clear need for future large-scale clinical studies to further examine such underlying pathophysiological mechanisms for a better understanding of such issues.

### Endoscopic bariatric interventions

Endoscopic interventions aimed at weight loss include endoscopic sleeve gastrectomy and band ligation, among others. There is more information on the effectiveness of such procedures on weight loss than on obesity-related complications.

Endoscopic sleeve gastroplasty is a potential alternative to sleeve gastrectomy [105]. A single-centre experience with 91 patients with a mean baseline BMI of 40.7 kg/m^2^ revealed a decrease in body weight (20.9% at month 24), haemoglobin A1c, SBP and serum triglycerides [106]. Such effects have been hypothesized to be mediated via increased GLP-1 levels, altered bile acid secretion, decreased serum ghrelin levels and improved peripheral insulin sensitivity [107, 108].

Intragastric balloons may be placed endoscopically and removed after 6 months but are potentially applied multiple times [109]. A meta-analysis including 10 randomized clinical trials and 30 observational studies with a total of 5668 patients undergoing intragastric balloon placement showed a statistically significant decreased DBP [mean −2.9 mmHg (95% CI −4.1 to −1.8)] despite non-significant differences in SBP compared with medical therapy [110]. Nevertheless, multiple large-scale randomized clinical trials have reported statistically significant decreases in SBP following intragastric balloon placement [111, 112]. Endoscopic gastric plication, gastric aspiration, duodenal mucosal resurfacing, endoscopic anastomosis systems and endoscopic bypass liners are other potential endoscopic bariatric procedures with limited clinical experience [113]. Even though initial results of endoscopic bariatric procedures on metabolic parameters, including SBP and DBP, are promising, there is a clear need for future large-scale clinical trials evaluating their long-term efficacy and safety for the management of obesity and obesity-related comorbidities.

## FUTURE PERSPECTIVES

Obesity and obesity-related comorbidities are major health concerns given their strong association with morbidity and mortality. GLP-1 receptor agonists are commonly prescribed antidiabetic and anti-obesity medications with potential beneficial effects on obesity-related comorbidities, including BP and hyperlipidaemia. More recently, dual or triple agonistic therapies activating GLP-1, GIP and glucagon receptors have emerged. Multiple ongoing clinical trials are investigating the efficacy and safety of such therapeutic approaches, including survodutide on body weight (NCT06066515, NCT06214741, NCT06492135) or obesity-related comorbidities such as cardiovascular events (NCT06077864); retatrutide on body weight (NCT05929066, NCT05929079) and cardiovascular outcomes (NCT06383390); and tirzepatide on body weight and obesity-related comorbidities (NCT06439277, NCT04255433, NCT05822830, NCT06075667, NCT05536804). Similarly, multiple ongoing studies have focused on endoscopic procedures for obesity and associated complications (NCT03705416, NCT05507151, NCT05739162, NCT04200144, NCT05917795, NCT04640688, NCT06339320). The outcomes of these ongoing clinical trials may potentially shed light on the optimal approach to treat overweight, obesity and their complications such as high BP.

**Figure 3:**
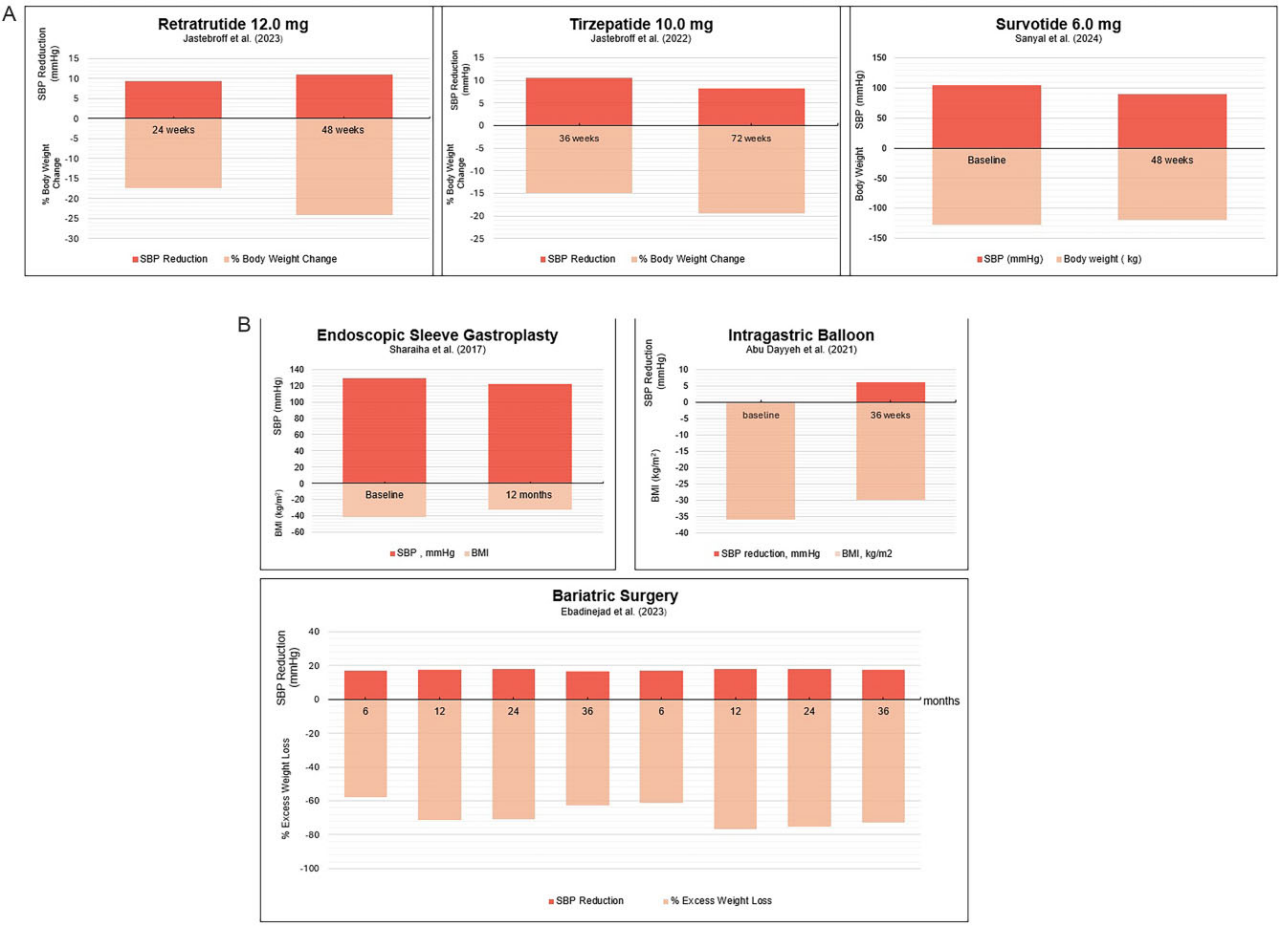
The potential role of various **(A)** pharmacotherapeutic and **(B)** interventional options for the management of obesity-related hypertension with respect to BP and body weight reduction.

There are few trials comparing the efficacy of bariatric surgery and pharmacotherapies for weight loss and obesity-related comorbidities, although medication has increased in popularity recently given that they are non-invasive, safe and efficacious (Fig. 3). A recent observational retrospective cohort study involving 3035 age- and gender-matched patients with a median follow-up period of 6.8 years undergoing either bariatric surgery or GLP-1 receptor agonist therapy did not observe advantages of either approach in terms of all-cause mortality or non-fatal MACE when adjusted for weight loss [114]. A meta-analysis summarizing data from 332 participants in six clinical trials concluded that bariatric surgery was superior to GLP-1 receptor agonist therapy in the population studied in terms of weight loss, although no differences in glycaemic control were observed [115]. A large-scale population-based retrospective cohort study demonstrated an advantage of bariatric surgery in terms of heart failure, cardiovascular events and cerebrovascular disease reduction over 3, 5, 7 and 10 years of follow-up versus GLP-1 receptor agonist therapy [116]. However, these long-term studies did not address the most recent GLP-1 receptor agonists, which have conclusively demonstrated cardiovascular and kidney protection. Similarly, metabolically associated fatty liver disease risk was significantly lower with bariatric surgery [117]. However, there are currently no clinical studies comparing the efficacy of either dual or triple agonist therapies with bariatric surgery for weight loss or obesity-related comorbidities. There is a clear need for future large-scale clinical studies evaluating which therapeutic approach works better for which patient, integrating baseline characteristics such as BMI and comorbidities, including BP control.

## Data Availability

No new data were generated or analysed in support of this research.
